# The World Health Organization's Rehabilitation 2030 vision: an African perspective

**DOI:** 10.3389/fresc.2024.1442626

**Published:** 2024-07-16

**Authors:** Maurice Douryang, Lervasen Pillay, Nonhlanhla S. Mkumbuzi, Calogero Foti

**Affiliations:** ^1^ Department of Physiotherapy and Physical Medicine, University of Dschang, Dschang, Cameroon; ^2^ Section Sports Medicine, Faculty of Health Sciences, University of Pretoria, Pretoria, South Africa; ^3^ Department of Sport, Exercise, and Rehabilitation, Northumbria University, Newcastle upon Tyne, United Kingdom; ^4^ Department of Human Movement Science, Nelson Mandela University, Qheberha, South Africa; ^5^ Department of Rehabilitation, Midlands State University, Gweru, Zimbabwe; ^6^ NtombiSport (PTY) Ltd, Cape Town, South Africa; ^7^ Physical and Rehabilitation Medicine, University of Tor Vergata, Rome, Italy

**Keywords:** WHO, rehabilitation 2030, health system, African region, strengthening

## Introduction

Rehabilitation 2030 is a World Health Organization (WHO) concept for the development of a new initiative and vision on rehabilitation. This has stemmed from the profound unmet need for access to rehabilitation services and research, also in the field of primary healthcare, for a range of disabling acute, acute-on-chronic, and chronic conditions worldwide (1). As part of Universal Health Coverage (UHC), rehabilitation is a key component of the healthcare system. Prioritising rehabilitation will reduce the burden of disability. The WHO aims to goad world leaders and stakeholders to strengthen their healthcare systems to provide high-quality rehabilitation services. The aims of the WHO initiative are being partially realised in many regions of the world, and most health-related issues involve rehabilitation. In Africa, the increase in the incidence of disability is alarming due to (1) traffic and workplace accidents, (2) complications of medical interventions, (3) natural disasters and conflicts, (4) poor access to education, (5) communicable diseases (e.g., malaria, poliomyelitis, and leprosy), and (6) non-communicable diseases (e.g., diabetes, hypertension, and cancer). This begs the question about the effectiveness of the present role of rehabilitation in Africa. Is Africa adequately prepared with qualified rehabilitation professionals and is there access to resources to achieve the WHO's rehabilitation action goals by 2030? To answer these questions, we need to identify African-specific challenges and should aim to address them.

## Highlighting the various challenges

### Socio-cultural habits, perception of disability, and rehabilitation

Disability is defined as a difficulty or inability to perform various activities in physical or mental functional domains. Examples of these are impairments in seeing, hearing, mobilising, memory, concentration, muscular strength, pain perception, self-care or communication, and problems such as anxiety and depression (1–4). However, these disability-related concepts are still poorly understood on the African continent. The reasons for this are not clear and may be attributed to different views on disability resulting from the cultural and social differences of various countries. An example of such differences can be found in the recent COVID-19 pandemic. Many African countries did not include rehabilitation in their COVID-19 management protocols even at a later stage as the evidence evolved (5). This was possibly due to the low primary care practitioner-to-patient ratio on the continent. Little attention was focused on the consequences of infection (e.g., post-COVID-19 fatigue, postpulmonary infection rehabilitation). Different countries in Africa have different healthcare approaches depending on the prevailing healthcare needs of the communities and access to resources.

In Cameroon, only three categories of disability are recognised: (1) physical, (2) mental, and (3) multiple disabilities, but these are not specified. Chad has specified visual and hearing disabilities for those categories of disability adopted by Cameroon. Disability always poses challenges of acceptance, adaptation, integration, and/or participation in Africa (4). This poor understanding of the condition imposes a burden on African communities and acts as a barrier to improve access to physical and mental rehabilitation.

Several African countries lack appropriate screening policies for disabilities among infants and children (e.g., cerebral palsy, congenital abnormalities) (6). This may be due to disability being perceived by many communities on the African continent as a curse, a manifestation of the forbidden, or an expression of punishment to the family or community (4). These African cultural concepts further burden those with disabilities and make them approach rehabilitation through traditional cultural methods rather than modern medical practices. However, as societies are increasingly exposed to a more Western-like healthcare system, they realise the value of rehabilitation.

This change in thinking will allow healthcare rehabilitation approaches to work in tandem with traditional approaches with a better likelihood of achieving the rehabilitation goals, as envisioned by the WHO, by 2030.

### Rehabilitation education within the health system

Education is a key strategy for advancing quality rehabilitation services worldwide. However, there is a paucity of physical and rehabilitation medicine curricula within the academic environment of the majority of African countries (4). Despite this fact, some universities and institutions do offer qualifications in fields such as physiotherapy, speech therapy, biokinetics, occupational therapy, orthotics/prosthetics, and psychology (3, 4). But these programs are limited in Africa. Undergraduate medical training curriculums leave clinicians underprepared in efficiently prescribing exercise-based rehabilitation. A healthcare practitioner interested in rehabilitation training will need to attend some courses or pursue postgraduate qualifications to develop exercise prescription skills. The present level of training focuses only on the prescription of common medications and not on lifestyle changes.

Therefore, it will be crucial and imperative to adapt healthcare practitioner training to include the use of exercise and rehabilitation as a primary and secondary prevention and treatment tool in Africa and promote the development of rehabilitation and movement sciences.

### Research and technology in rehabilitation

Rehabilitation research is limited in Africa compared with other regions in the world. Research performed on other continents with different resource availabilities and accesses cannot be duplicated in all countries of the African continent because of the heterogeneity of professionals available and resources that can be accessed. There needs to be an African solution for an African problem—which will be more conducive to evidence-based practice implementation and dissemination on the African continent. Africa has obstacles not identified by other types of research in other countries (4). Some parts of Africa have barriers to including technology in rehabilitation. These include, amongst others, (1) education on its use and the significance of its findings, (2) cost factors for access, (3) affordable access to the internet, and (4) socio-cultural effects. Benefits may be had in developing an African Rehabilitation Council for all African countries. Strategically teaming with international collaborators would prove advantageous to the continent in terms of sharing knowledge.

Advances in technology, such as in the fields of artificial intelligence and telemedicine, appear to offer potential opportunities to bridge rehabilitation gaps and enable good strategies for expanding assisted and remote rehabilitation. However, the reliance of these technologies on internet connectivity may prove to be a challenge in some countries in Africa.

For these reasons, Africa needs to embrace technological innovation to advance rehabilitation by mobilising the necessary resources. Developing a disability map to identify areas that require rehabilitative services may help in implementing strategies.

### Poverty and health system financing

High-quality rehabilitation services are costly due to the cost of treatments, the use of equipment, and the time spent on the rehabilitation process (3). While the majority of the African population face the challenge of extreme poverty and are devising ways and means to overcome this challenge almost on a daily basis, they do not pay attention to their medical expenses (including rehabilitation expenses), and as a result, these tend to be neglected (7).

Survivors from conflicts or other medical-related issues are often left with long-term disability. This adds to further medical costs and economic strain on families and communities. These financial challenges may be the reasons why the issue of rehabilitation is approached in traditional cultural ways rather than from a medical perspective.

## Discussion

The leadership and governance of the national healthcare systems of African countries focus on financing disease treatment via medication only when healthcare system infrastructure and the development of disease prevention strategies are overlooked (8). However, prevention should be one of the main ways to tackle non-communicable diseases and rehabilitation should be done after one contracts a communicable disease. These are of particular value in a low-income country where there is healthcare resource limitation. Health insurance is a luxury for many Africans. Contrastingly on other continents, strategies have been formulated for providing health cover for all without any discrimination (7). With most Africans either employed in the informal economic sector or unemployed, it would be imperative to include the costs of the different aspects of rehabilitation if individual governments intend providing health insurance, which will help further increase the relevance of Universal Health Care. A discussion on how to overcome existing financial barriers and expand health insurance coverage for all on the African continent is beyond the scope of this article, but any discussion that deals with the aforementioned points and helps improve access to appropriate rehabilitation measures for the population will always be useful at any given time.

Therefore, community-based rehabilitation programs can be developed for different conditions prevailing in this country, but they need to be supplemented by effective governance, available resources, expertise, and community participation.

### Physical activity/exercise and prevention of chronic diseases

Non-communicable diseases in Africa highlight shortcomings in health systems both at a social and at a welfare level (8). Physical activity is an appropriate (9), cost-effective, and evidence-based strategy to prevent and manage chronic diseases and promote health. Healthcare professionals involved in this field should be encouraged to join and develop effective nationally led systems and must be supported through policy so that these strategies could become effective.

## Proposal

### A call to action

With the 10-point call to action statement that we have suggested in Table 1, we are confident that countries in Africa can improve the response to disabilities and undertake appropriate rehabilitation.

**Table 1:** Ten key strategic actions for Africa to achieve the WHO Rehabilitation 2030 goals.

	Actions	Description
1.	Optimising rehabilitation education	Undertaking implementation and dissemination to include rehabilitation in healthcare professionals’ curricula across Africa. Assuring training of all rehabilitation professions, including the specialty of physical and rehabilitation medicine. Sensitising other practitioners about the capabilities of other rehabilitation professions in an African context via online meetings and conferences. Continuous updating of other healthcare providers on rehabilitation services. Promoting remote rehabilitation via technology. Promoting the use of postgraduate courses and qualifications to implement lifestyle changes.
2.	Financing rehabilitation research	Funding for the strengthening of rehabilitation research to high-quality scientific production and practical evidence. Supporting research for technological innovation to improve assisted rehabilitation. This should involve African populations on the African continent.
3.	Creating a disability map	Improving surveillance and monitoring of disability through area visits to obtain a disability map for providing efficient rehabilitation service approaches for different conditions in different countries.
4.	Improving the integration of rehabilitation in health systems across Africa	Integrating rehabilitation into primary care. Facilitating access to rehabilitation services by expanding rehabilitation structures and accessibility across countries on the continent
5.	Implementing and disseminating adequate rehabilitation infrastructures	Improving and expanding specialised rehabilitation infrastructures by improving equipment and internet access for remote rehabilitation
6.	Reorganising the policy on cost for rehabilitation care	Including rehabilitation for appropriate conditions in healthcare costs. Establishing rehabilitation care in all countries in Africa. Implementing an awareness campaign on the benefits of exercise and rehabilitation for targeted medical conditions.
7.	Improving governance actions with regard to the benefits of rehabilitation services	Multi-sectorial collaboration is needed: The government, stakeholders in rehabilitation, and organisations must design effective evidence-based recommendations on disability and rehabilitation actions that are country-specific. Health[M V20] departments should update the definition of disability categorisation and then raise public awareness to inform who has access to rehabilitation services. There must be interaction between Disability and Rehabilitation Divisions within countries’ Departments of Health.
8.	Strengthening national and international partnerships and frameworks for rehabilitation development	Establishing partnerships with other rehabilitation experts who have experience in implementing rehabilitation in different contexts worldwide. Setting up a strong continental and national taskforce to tackle disability-related issues.
9.	Promoting exercise-based rehabilitation for disease prevention	Implementing a policy to promote physical activity for disease prevention. Encouraging sport participation as an activity or profession in both able and physically challenged people. Creating facilities to promote physical activities.
10.	Promoting strong media communication on disability and rehabilitation	Communicating widely about disability to dispel ignorance and avoid prejudice and stigmatisation. Strong media communication about rehabilitation services and their benefits and role must be promoted.

### Take-home message

The development of rehabilitation care is challenging worldwide. Africa has additional complex contextual barriers to the implementation and expansion of rehabilitation. These include a lack of educational programs, limitation of rehabilitation integration in healthcare systems, poverty, difficulties in accessing rehabilitation in primary care, misperception of disability and rehabilitation, lack of a disability map, limited research funding, poor or absent policies, and poor government support. To significantly contribute to the future of rehabilitation as expected by the WHO, Africa needs to take concrete actions such as those suggested in our proposed 10-point Action Plan.
